# Ozone pollution contributes to the yield gap for beans in Uganda, East Africa, and is co-located with other agricultural stresses

**DOI:** 10.1038/s41598-024-58144-1

**Published:** 2024-04-05

**Authors:** K. Sharps, J. Foster, M. Vieno, R. Beck, F. Hayes

**Affiliations:** 1 UK Centre for Ecology & Hydrology, Environment Centre Wales, Deiniol Road, Bangor, Gwynedd LL57 2UW UK; 2 UK Centre for Ecology & Hydrology, Bush Estate, Penicuik, Midlothian EH26 0QB UK

**Keywords:** Environmental impact, Atmospheric chemistry, Abiotic

## Abstract

Air quality negatively impacts agriculture, reducing the yield of staple food crops. While measured data on African ground-level ozone levels are scarce, experimental studies demonstrate the damaging impact of ozone on crops. Common beans (*Phaseolus vulgaris*), an ozone-sensitive crop, are widely grown in Uganda. Using modelled ozone flux, agricultural surveys, and a flux-effect relationship, this study estimates yield and production losses due to ozone for Ugandan beans in 2015. Analysis at this scale allows the use of localised data, and results can be presented at a sub-regional level. Soil nutrient stress, drought, flood risk, temperature and deprivation were also mapped to investigate where stresses may coincide. Average bean yield losses due to ozone were 17% and 14% (first and second growing season respectively), equating to 184 thousand tonnes production loss. However, for some sub-regions, losses were up to 27.5% and other crop stresses also coincided in these areas. This methodology could be applied widely, allowing estimates of ozone impact for countries lacking air quality and/or experimental data. As crop productivity is below its potential in many areas of the world, changing agricultural practices to mitigate against losses due to ozone could help to reduce the crop yield gap.

## Introduction

Africa experiences some of the worst air pollution and health consequences in the world—in 2019, air pollution was the second leading risk factor for death across Africa after malnutrition^1^. Tropospheric (ground-level) ozone (O_3_), is a harmful air pollutant for human health and vegetation, causing reductions in quality and quantity of crop yield in sensitive species around the world ^2–4^. Ozone is formed when precursor gases including volatile organic compounds (VOCs), nitrogen oxides (NOx), carbon monoxide and methane, primarily from anthropogenic sources such as emissions from vehicles and industry, react together in the presence of sunlight^5^. In Africa, measured ozone data are generally sparse, however, South African monitoring stations have regularly measured tropospheric ozone levels above the air quality standard limit^6^. Ambient ozone concentrations were measured using 120 diffusion tubes across Uganda in Dec. 2018–Feb. 2019^7^. Mean values ranged from 32 to 88 ppb and were highest in smaller urban centres and rural areas. Ozone concentrations are projected to rise further in many developing regions^8^. An increase in surface mean ozone concentrations by 2100 is predicted for parts of Africa, particularly around the equator, due to projected changes in climate^9^.

Experimental and modelling studies have highlighted the potential negative impact of ozone on the yield of African crops. For example, elevated ozone levels led to a reduction in 100-seed weight and seeds per pod for cowpea (*Vigna unguiculata* L.)^10^. Reductions in total yield and 1000-grain weight have been demonstrated for African cultivars of common wheat (*Triticum aestivum*) and common bean (*Phaseolus vulgaris*) in high treatment levels of ozone (mean daily max concentration of 84 ppb and 93 ppb, respectively)^11^. Sweet potato also showed a ~ 40% and ~ 50% reduction in yield at medium (80 ppb) and high (110 ppb) levels of ozone respectively^12^. Using modelled ozone data for Sub-Saharan Africa, estimated losses for common bean were > 20% in some countries, including Uganda^13^. Analysis at a finer scale allows more localised data to be included, and results can be presented at a sub-regional level, better highlighting locations at high risk from ozone impacts on crop yield.

Uganda, in central east Africa, is highly dependent on agriculture^14^. Country-wide agricultural surveys were carried out in 2008/2009, and 2018^15,16^. Beans are widely grown on a subsistence level and are an important source of protein. The Ugandan bean production system is predominantly small-scale, with average production (250 kg/acre) being low compared to potential yield (700 to 1500 kg/acre) ^17^. Ugandan crop production is characterised by low input use and the majority of bean farmers use non-commercial seed from previous harvests^18^. Improved Ugandan bean varieties have been developed by NARO (National Agricultural Research Organisation), however, these often do not reach farmers due to underfunded extension services and for those that do, they may be rejected due to their incompatibility with local farming systems^19^.Ugandan bean production increased considerably between 2000 and 2010^16^, which was associated with increasing population^20^. FAO (Food and Agriculture Organisation of the United Nations) national production totals suggest that levels continued to increase between 2010–2017 but were beginning to level out^21^ and by the next nationwide agricultural survey in 2018 levels had begun to decline^16^ Between 2010–2016, bean exports from Uganda increased from 2.5 to 26% due to rising demand^17^, with bean consumption in East Africa increasing yearly between 2006 and 2016^22^. If exports continue to increase without a corresponding increase in yield, this could have negative impacts as farmers also rely on beans for their own households. Different areas of Uganda experience varying levels of surplus and deficit of bean production^18^, for example, the sub-regions of Acholi, Lango and Teso, in the north and east are mostly self-sufficient in bean production, with some areas of minor deficit. The eastern sub-region of Karamoja has a deficit of bean production as this area is prone to regular droughts, conflicts and high poverty levels.

Bean farmers in Uganda face many constraints, including the cost of seeds^23^ and unstable prices^17^. Uganda is a low-income country and historically has been classed as one of the poorest countries in the world^24^. The country is also experiencing increased frequency and severity of extreme weather events^25^, for example, droughts, floods and increases in temperature, which can reduce yield and lead to crop losses before maturity^26–29^. The majority of Ugandan crop production depends on seasonal rainfall, which results in a vulnerability to changes in climate^30^. Bean production is also adversely affected by waterlogging of soil due to periods of heavy rain^31^. Further challenges include declining soil fertility^17^. In Uganda, a considerable proportion of soils are highly weathered, and have low nutrient reserves, therefore low capacity to supply nutrients to crops^32^. Air pollution is a further contributor to the bean yield gap in Uganda, with potential interactions possible between drought and ozone exposure. As drought leads to stomatal closure, ozone impacts can be reduced under drier conditions for some crop species^33,34^. This is a complex issue however and is not the case for all plants^35^. In turn, the irrigation of crops can lead to an increase in ozone uptake (also referred to as ozone flux) into the leaves, leading to further yield losses^2^. Investigating how factors impacting crop yield, including ozone pollution, nutrient stress, drought, floods, temperature and deprivation/ poverty occur spatially across Uganda would highlight hotspots where these stresses coincide, indicating areas at particularly high risk of yield constraints.

This study will bring together modelled ozone data (from the European Monitoring and Evaluation Programme (EMEP) chemical transport model), up-to-date spatial data on African crop production (Spatial Production Allocation Model (SPAM) 2017), Ugandan agricultural survey data^15,16^, detailed information on growing seasons and a breakdown of production per sub-region to estimate yield and production losses due to ozone for common beans in 2015. A species-specific flux effect relationship for widely grown bean cultivars from experimental data will be used to provide estimates of yield losses due to ozone^11^. Flux-based models have been found to perform better than concentration-based models^36^ and to provide improved predictions of the distribution of ozone damage^37^. Spatial data on nutrient and drought stress, flood occurrence, temperature and levels of multidimensional deprivation will also be mapped to investigate areas in the country where crop stress factors are at their highest. The use of fine scale and localised data will provide more robust estimates of potential losses due to ozone, compared to larger scale studies, for an important food crop in a country dependent on agriculture.

## Materials and methods

### Study area

**Figure 1:**
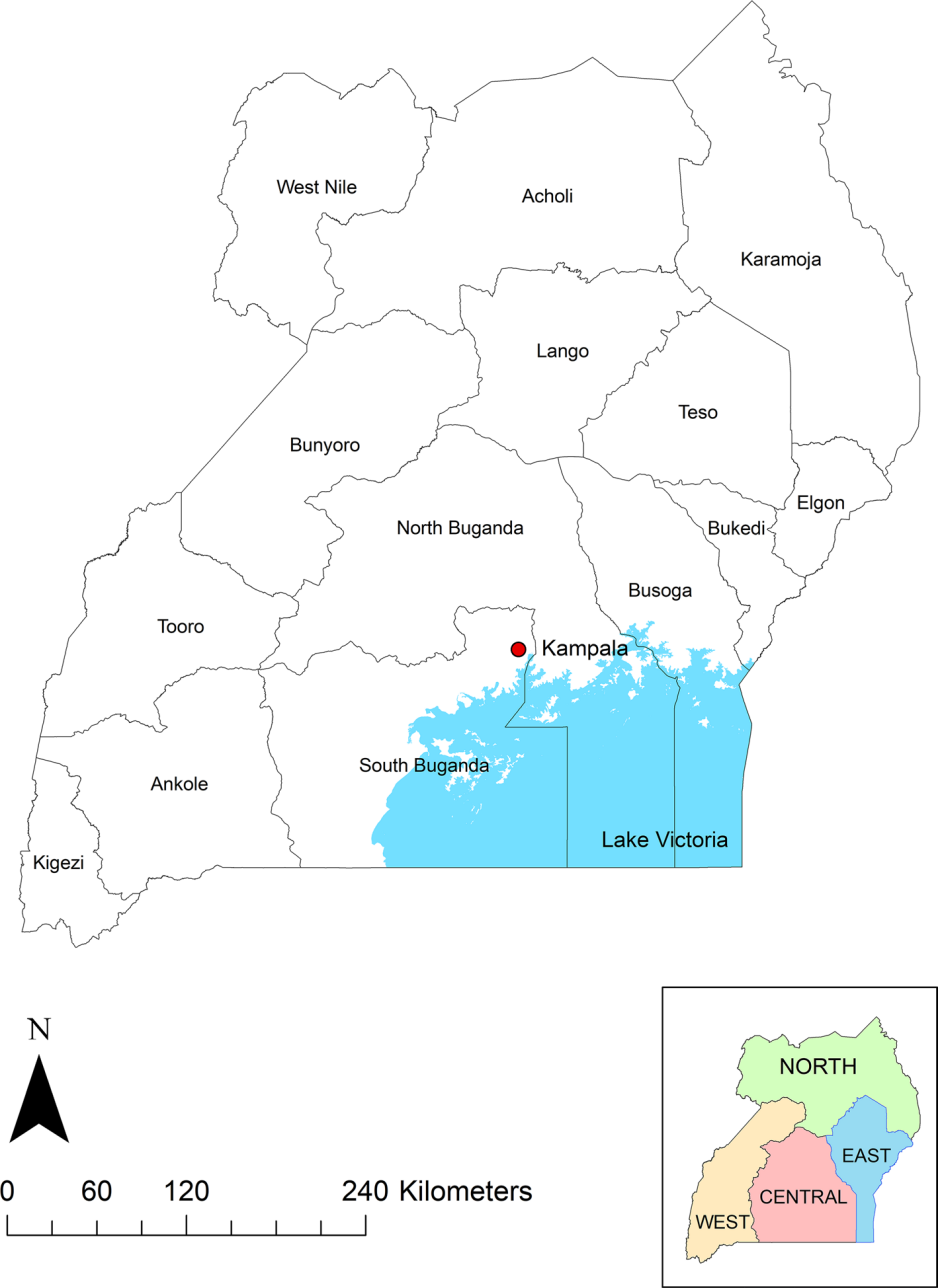
Map of Uganda, split into 14 sub-regions. The capital city, Kampala, is shown as a red point, also included are Lake Victoria (coloured blue), and as an inset, the 4 regions of the country (Northern, Western, Central and Eastern). The map was generated using ArcMap (version 10.6), https://www.arcgis.com.

Uganda has central, western, eastern and northern regions and 14 sub-regions (Fig. 1). The Annual Agricultural Survey (AAS) (2018) survey report^16^ provides seasonal crop production data for the 14 sub-regions. In this study, ozone impacts on bean production will be reported at the sub-region level.

### Modelled ozone data

The ozone flux metric, POD_3_IAM (phytotoxic ozone dose above 3 nmol m^−2^ s^−1^, parameterised for integrated assessment modelling), was chosen as the most suitable for this study as it has a lower influence of phenology on ozone uptake (compared to the species-specific POD_6_SPEC metric), leading to lower reliance on the precise timing of local crop growth times. Data were produced for 2015 by the EMEP-WRF Africa model, version 4.33^38^. This model is based on the official EMEP MSC-W (European Monitoring and Evaluation Programme, Meteorological Synthesising Centre-West) chemical transport model^39^, which has been used in air quality assessments for more than 30 years. The EMEP-WRF Africa model uses meteorological data from the Weather Research and Forecast (WRF) model^40^. The model domain covers the globe with a horizontal resolution of 1.0 × 1.0° and a nested domain covering the African continent with a horizontal resolution of ~ 0.33 × 0.33° (~ 37 × 37 km at the equator). The emissions used were based on the International Institute for Applied Systems Analysis (IIASA) ECLIPSE v6a (Evaluating the Climate and Air Quality Impacts of Short-Lived Pollutants) GAINS (Greenhouse gas–Air pollution Interactions and Synergies) model for the year 2015^41^.

The EMEP-WRF Africa model outputs daily values for ozone surface concentration (ppb). The DO_3_SE (Deposition of O3 for Stomatal Exchange) model^42,43^ is embedded within the EMEP model, and calculates ozone flux, using data on environmental factors (including light, temperature, vapour pressure deficit), which can influence the opening and closing of stomata, and thus ozone uptake^13^.

The ozone flux metric POD_3_IAM was calculated with the EMEP model parameterised for integrated assessment modelling (IAM). This involves using simplified flux models for a vegetation type (e.g. crops) rather than one species. The EMEP model outputs irrigated (calculated without soil moisture limitation) and non-irrigated (calculated using a modelled soil moisture index) POD_3_IAM values per grid cell. For this study, only non-irrigated POD_3_IAM data were used, as the majority of bean crops in Uganda are not irrigated.

The EMEP-WRF Africa model was parameterised using the POD_3_IAM crop inputs (based on European wheat), but these were adjusted for African beans using published data from experiments carried out on African crops in the UKCEH Bangor solardomes 2017–2019^11,44^. See Supplementary Information, Tables S1 & S2 for the model input values.

When working with chemical transport models, it is important to ensure that modelled values represent the atmosphere as adequately as possible, therefore outputs are often compared with measurements in a validation process (See Supplementary Information Sect. 2, including Fig. S1).

### Bean production in Uganda

Ugandan growing seasons are similar in most sub-regions across the country. Variations in agro-ecological zones impact the type of crops grown more than the harvest dates. In general, season 1 harvest is in June/July and season 2 harvest is in November/December. The exception to this is in Karamoja in the northeast of Uganda (Fig. 1). Unlike the rest of the country, Karamoja has only one key growing season, due to a unimodal rainfall pattern^18^, with harvest dates from August to November. (See Supplementary Information, Table S3).

Spatial data for bean production in Uganda were derived from the Spatial Production Allocation Model (SPAM) dataset for 2017, at a resolution of 0.0833° × 0.0833° (approx. 10 km at the equator)^45,46^. SPAM data is given as an irrigated and non-irrigated, i.e. rainfed, production total for each grid cell. As irrigated production values were zero for all cells in 2017, only non-irrigated production data were used.

Regional conversion factors were applied to the 2017 bean production dataset to calculate estimated production values for 2015. These conversion factors were calculated by determining how regional production quantities had changed between the 2008/2009 and the 2018 agricultural surveys. For further details, see Supplementary Information, Table S4.

Using ArcMap (version 10.6), a 0.0833° × 0.0833° grid (aligned with the SPAM dataset) was created, and for each cell, the bean production and sub-region where the cell was located were determined.

### Calculating 90-day POD_3_IAM values

To encapsulate the time when crops are most sensitive to ozone, the daily POD_3_IAM data were summed over a 90-day period before harvest. This period includes the ozone-sensitive period between anthesis and the end of grain fill^47^. For all bimodal sub-regions, the 90-day period used for season 1 spanned from April to June (day 91–180, Julian calendar), and for season 2 spanned mid-Sep to mid-Dec (day 257–346). For Karamoja, the 90-day period used was July to September (day 182—271). Data sources for Ugandan growing periods included crop calendars from FEWSNET (Famine Early Warning Systems Network; https://fews.net/ ) and the FAO.

### Yield and production loss due to ozone

Percentage yield loss for beans due to ozone was calculated (Eq. 1). This flux-effect relationship is the first to be developed for common bean and was derived from experimental data, using cultivars widely grown around the world, including three grown in Kenya and one grown in South Africa (See Fig. S2). Comparison with a larger 7-h mean ozone dataset for legumes from the ICP Vegetation ‘crop sensitivity to ozone’ database suggests that the ozone sensitivity of the cultivars used in the ozone flux-response relationship is typical for cultivars grown around the world (Supplementary Information, Fig. S3). Generalised least squares (gls) regression (using the R ‘nlme’ package^48^) was used to model the relationship between relative yield and POD_3_IAM, to allow for spread in the data as POD_3_IAM increased. The reference value was calculated for the experimental conditions under which the flux-effect relationship data were collected, assuming constant 10-ppb ozone across the 90-day period. For the bean experiment, this reference value was zero. Quantification of the level of risk is based on the slope of flux-effect relationship, without taking the intercept of the response function into account^49^. In the case of the bean flux-effect relationship, this can be justified by the slope of the response regression being strongly significant (*p* < 0.001), the 95% confidence limits of the intercept including 100 and it is also generally relevant to assume that zero exposure is associated with zero effect.

1 $$\% Yield\, Loss = POD_{3}IAM \,*\, 1.175$$

Total production loss (Eq. 2) was calculated using the yield loss percentages with the estimated crop production values for 2015.

$$Production\, Loss = \left( {total \,production / relative \,yield} \right) - total \,production$$

2 $$\mathrm{where }\,Relative \,Yield=1/(\mathrm{\%}yield \,loss / 100)$$

The SPAM crop production data represent an annual total, so the data have been split between season 1 and season 2. For beans, approximately 60% of total production is harvested in season 1 and 40% in season 2^16^. The sub-regional breakdown can be seen in Supplementary Information (Fig. S4). A separate analysis was run for the unimodal sub-region of Karamoja.

Yield loss estimates were mapped for all 0.083° × 0.083° cells with data on bean production, (even if production = zero) to highlight areas that have the potential to be at risk of high losses due to ozone. Production losses due to ozone were calculated and mapped for grid cells with bean production > 0 for the study year.

### Additional crop stressors

To investigate areas where bean farmers may face multiple stresses, data were gathered on other possible factors which could impact crop yield. The first growing season was focused on, as this is when the majority of bean production occurs. For soil nutrient stress, classes for soil nutrient availability and nutrient retention were downloaded from the Global Agro-Ecological Zones (GAEZ) data portal (https://gaez.fao.org/) and combined to produce five soil nutrient stress classes, ranging from ‘No constraints’ to ‘Very severe constraints’ ^50^ and mapped for Uganda, at 0.083° by 0.083° resolution. The soil classes have been derived from combinations of soil attributes, using data in the Harmonized World Soil Database (HWSD, v. 1.1, FAO/IIASA/ISRIC/ISS CAS/JRC 2009). As a measure of drought stress, the Standardized Precipitation-Evapotranspiration Index (SPEI) dataset was used, which has been computed monthly for Africa at 5 km resolution, for the period 1981 to 2016^51^. SPEI is calculated using data on long-term precipitation and atmospheric evaporative demand. Values range from Extremely Wet (2 and above) to Extremely Dry (-2 and less). To facilitate the diagnosis of droughts of different durations, accumulation periods from 1 to 48 months are provided^51^. The memory of moisture conditions in previous months can be accumulated to later months^52^. The mean SPEI value per 0.083° grid cell was calculated. For the current study, an accumulation period of 6 months was used, for the year 2015, with the average value calculated per grid cell for April to June. Flood occurrence data for each Ugandan district^53^, which were collated from data repositories including Dartmouth Flood Observatory^54^, DesInventar^55^ and the Emergency Events Database (EM-DAT)^56^ were mapped for 2007–2015, providing a measure of flood risk. Where Ugandan grid cells spanned multiple levels of flood risk, the cell was designated the level of flood risk with the greatest area. Monthly temperature data (mean daily maximum, °C) were downloaded from WorldClim (https://www.worldclim.org), which are Climatic Research Unit gridded (0.1667°) Time Series data (CRU-TS 4.06)^57^, downscaled using WorldClim2^58^. A mean was calculated per 0.083° grid cell across Uganda for the months of May and June 2015. Common bean grows well at temperatures ranging from 20 to 28 °C and can withstand temperatures up to 29.5 °C, however high temperatures (close to or higher than 35 °C) during flower and pod setting (6–8 weeks after planting) result in abortion of large numbers of blossoms and developing pods^17,59^.

Lastly, the Global Gridded Relative Deprivation Index (GRDI; v1), from NASA’s Socioeconomic Data and Applications Centre (SEDAC) was mapped across Uganda, characterising the relative levels of multidimensional deprivation and poverty, at a resolution of 30 arc-seconds (~ 1 km grid cells)^60^. The inputs for the GRDI dataset represent six dimensions of deprivation—child dependency ratios, infant mortality rates, a subnational human development index, building footprints per square kilometer, and night-time lights^61^. The index ranges from 100 (highest level of deprivation) to 0. The mean deprivation index value was calculated per 0.083° grid cell.

These measures of crop stress, along with the ozone yield loss dataset for season 1 were scored from 1 to 5 (very low to very high risk to crop yield) and scores were summed to calculate a ‘crop stress score’ per 0.083° grid cell. The scoring method for ozone and nutrient stress followed previously published methodology^50^. See the Supplementary Information (Table S5) for further information on the scoring and Fig. S5 for a flowchart showing the key steps of the methodology used in this study.

### Human or animal rights

No animals, human subjects or human data were used in the study.

## Results

### Surface ozone levels

**Figure 2:**
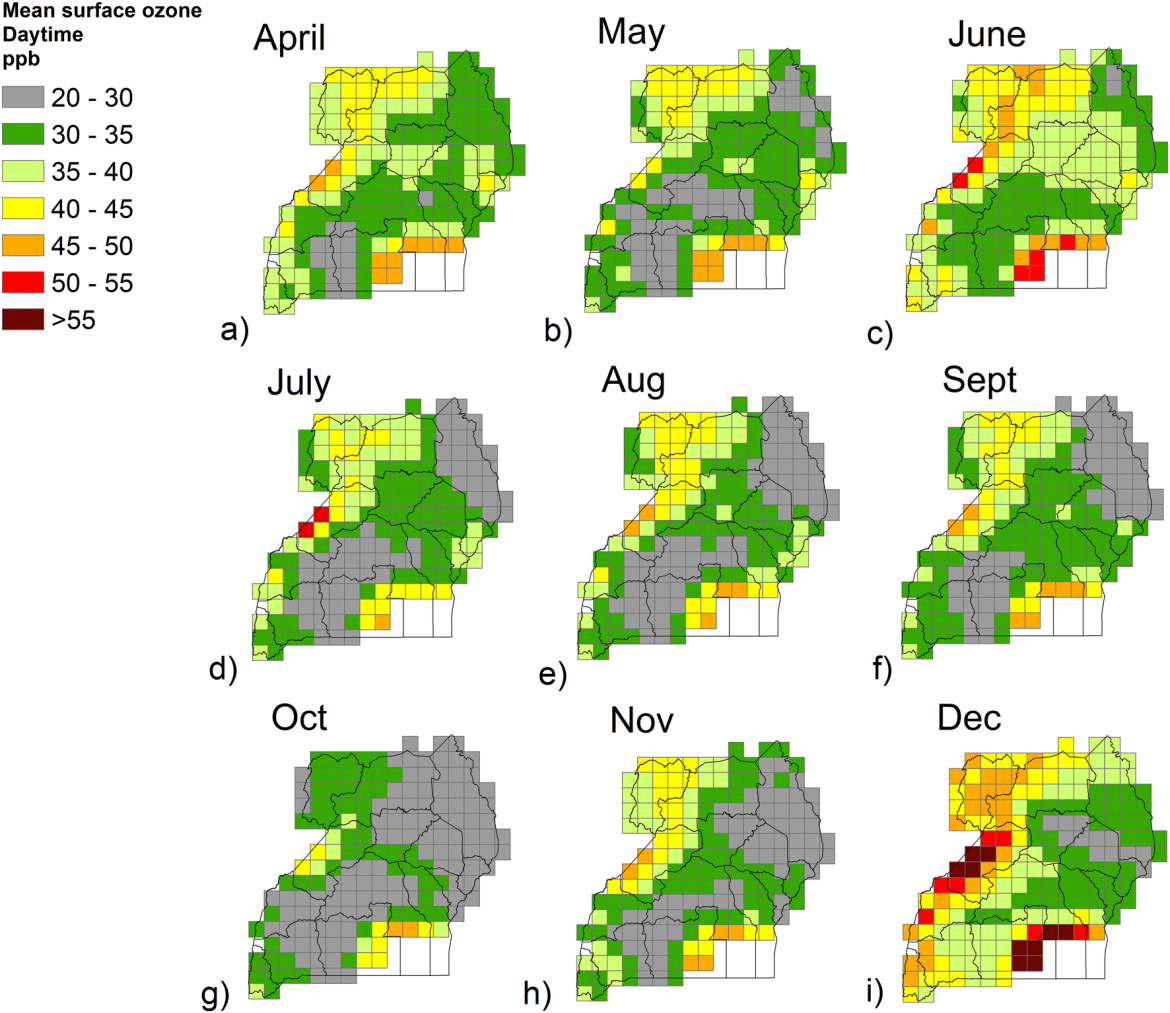
Modelled daily mean surface ozone concentration (ppb), per month, for Uganda in 2015. Modelled data are from the EMEP-WRF Africa model and daily mean values are for the period 6am–6am, averaged for each month (**a**–**i**) of the Ugandan bean growing season (April to December). The maps in the figure were generated using ArcMap (version 10.6), https://www.arcgis.com.

Modelled ozone levels for 2015 varied with time and location (Fig. 2). Daily mean values (for the period 6am – 6 pm) averaged for each month ranged from 23 to 62 ppb. Surface ozone tended to be higher in the north-west of the country, while levels were lower in the eastern, south-west and central areas. The month with the lowest surface ozone overall was October, and values began to increase again later in the year, with the highest levels seen in December (> 55 ppb in Bunyoro and > 50 ppb in the Tooro sub-region). There were also higher values estimated in the south, along the coast of Lake Victoria, however, beans are not grown there.

### Bean production

**Figure 3:**
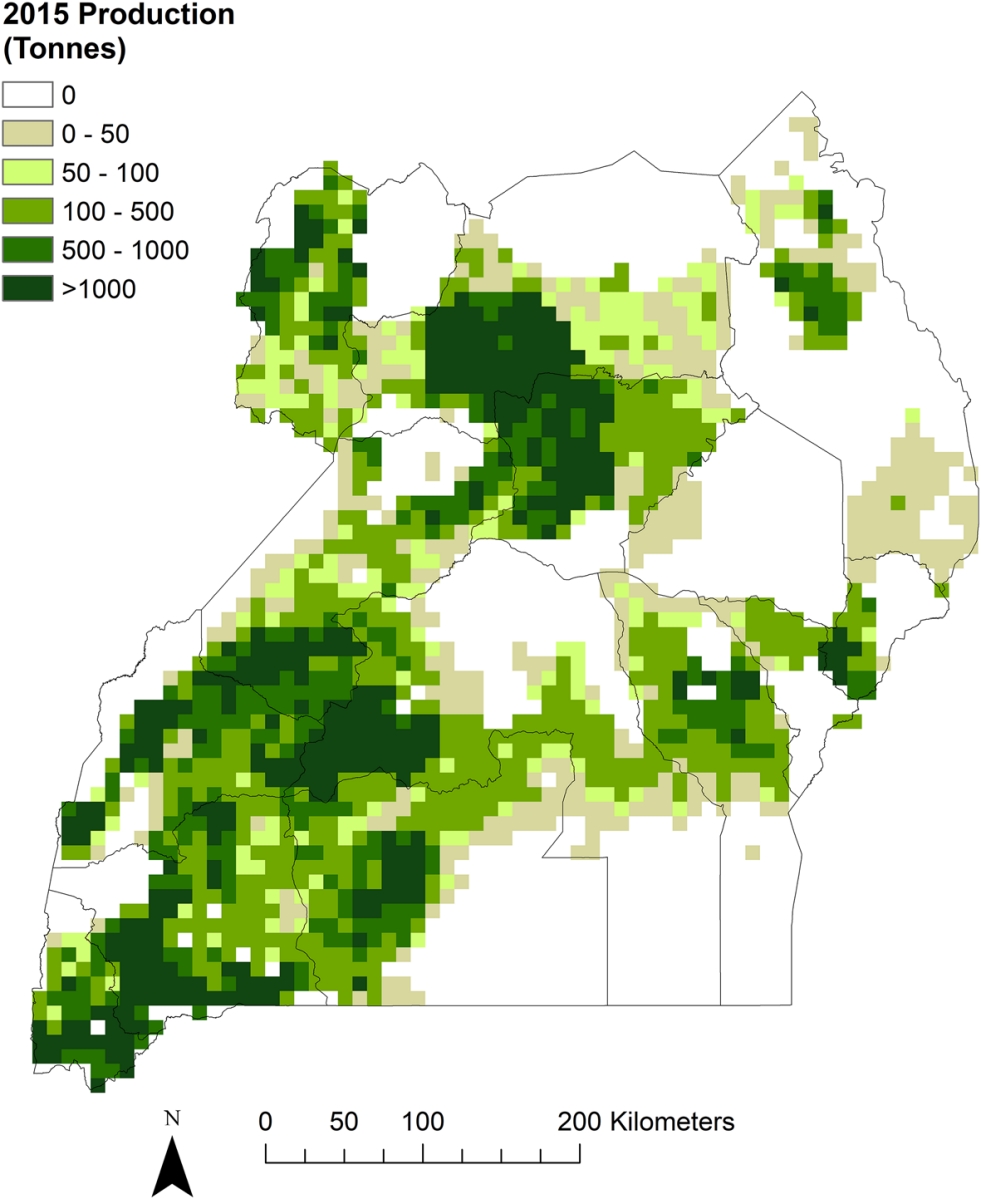
Total annual production of common beans (*Phaseolus vulgaris*) across Uganda in 2015. Model results from the Spatial Production Allocation Model (SPAM) for the year 2017 have been converted to 2015 values. Spatial resolution 0.0833° × 0.0833°. The map was generated using ArcMap (version 10.6), https://www.arcgis.com.

The highest production of beans in Uganda in 2015 (based on estimates using SPAM data for 2017), was in the Ankole sub-region in the south-west of the country, with a total of over 250,000 tonnes (Fig. 3, Table S6). Other high producing sub-regions included Acholi and Lango in the north, Tooro in the west and North Buganda in the central region. Some sub-regions had similar production levels in each season, for example, Ankole (Table S6), whereas Bunyoro and Busoga for example, had considerably more bean production in the first growing season than the second, and West Nile had more production in the second season (Figure S4, Table S6). Overall, regional production was highest in the west, followed by the northern and central regions, and lowest in the east. For comparison, production estimates from the 2008/9 and 2018 Annual Agricultural Survey are also given (Table S7). At the regional level, total production values are on a comparable scale for SPAM and survey data for the central, eastern and western regions, and the general trends for increasing values in the central region and decreasing values in the western region are also seen. However overall, the data suggest that estimates from the modelled 2017 SPAM dataset, particularly for the northern region, are higher than might be expected, compared with the 2018 survey data.

### Yield loss due to ozone

**Figure 4:**
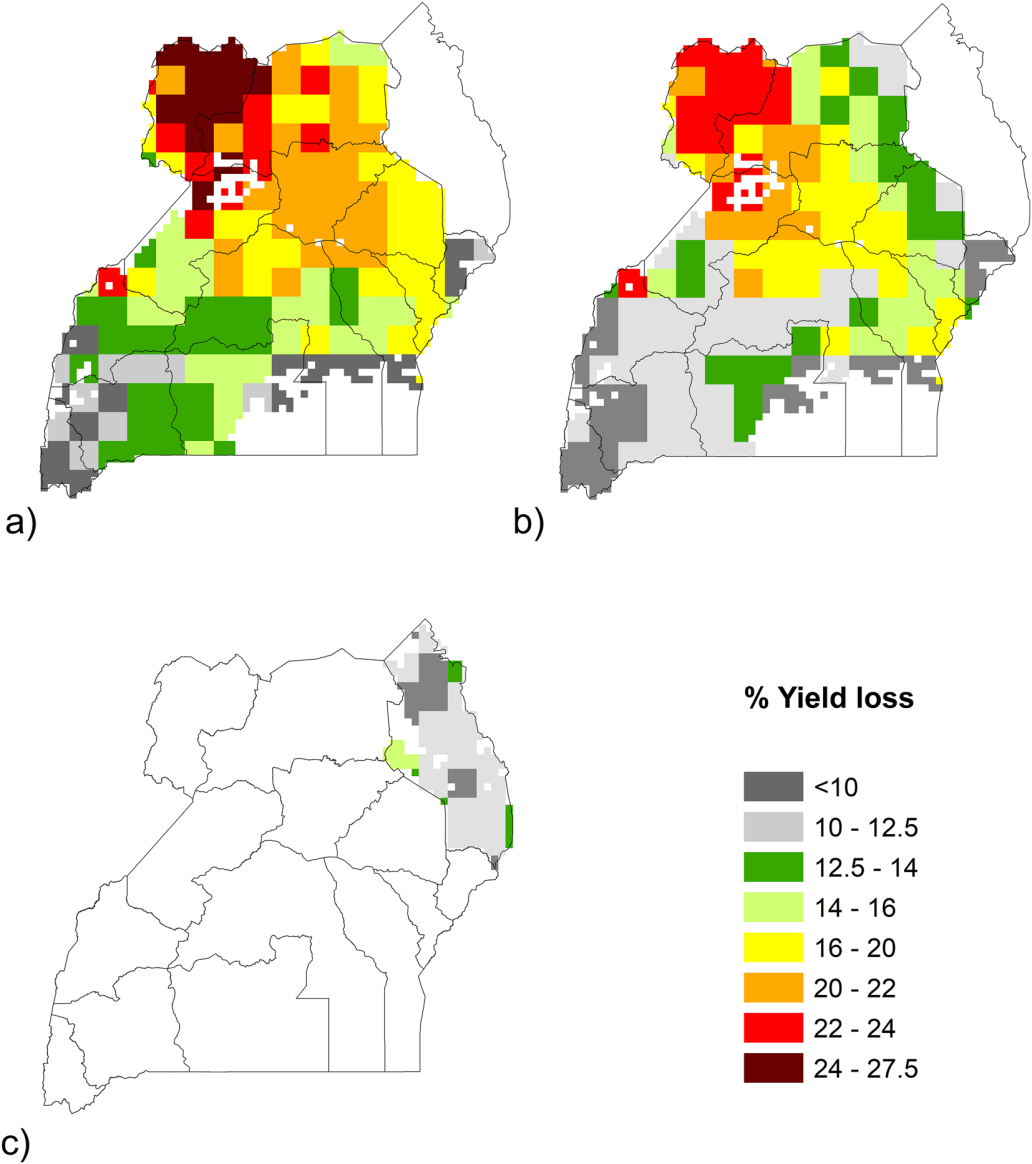
Percentage yield loss due to ozone for Ugandan beans in 2015. For (**a**) growing season 1 (April – June); (**b**) season 2 (mid-Sep to mid-Dec) and (**c**) the unimodal Karamoja sub-region (growing season July to Sep), using 0.083° × 0.0833°grid cells. Blank cells show areas with no data on bean production and/or POD_3_IAM = 0. Ozone data from the EMEP-WRF Africa model is 0.3° × 0.3° resolution. The maps in the figure were generated using ArcMap (version 10.6), https://www.arcgis.com.

Bean yield loss due to ozone varied depending on location, ranging from < 10 to 27.5% (Fig. 4, Table 1). Estimates also varied with season, with percentage yield losses being lower for season 2 than in season 1. In season 1, the highest estimates were in the north-west of the country, with an average yield loss of 24% in the West Nile sub-region (Fig. 4, Table 1). Yield losses were lower in southern and eastern areas, with the lowest yield losses estimated at 10% for the Elgon sub-region in the east and 8% for Kigezi in the south. In season 2, the highest losses were in the north-west (average of 20% for West Nile), and values were lower in central, southern and eastern areas, with the lowest values again in Elgon and Kigezi (7%). For Karamoja, the estimates for yield losses due to ozone during the single growing season ranged from 7 to 15% (average 11%).

**Table 1:** Average percentage yield loss due to ozone for beans per sub-region in Uganda, 2015.

Sub-region	Region	Average % yield loss	
		Season 1	Season 2
North Buganda	Central	15.61	13.86
South Buganda	Central	13.45	11.73
Teso	Eastern	18.87	14.26
Bukedi	Eastern	17.37	15.24
Busoga	Eastern	15.70	14.47
Elgon	Eastern	10.42	7.18
West Nile	Northern	23.93	19.86
Acholi	Northern	21.05	15.92
Lango	Northern	20.56	16.51
Karamoja	Northern	10.96	NA
Bunyoro	Western	18.56	15.77
Tooro	Western	13.65	11.27
Ankole	Western	12.02	10.14
Kigezi	Western	8.95	7.16
Average		17.09	14.14

### Production losses due to ozone

**Figure 5:**
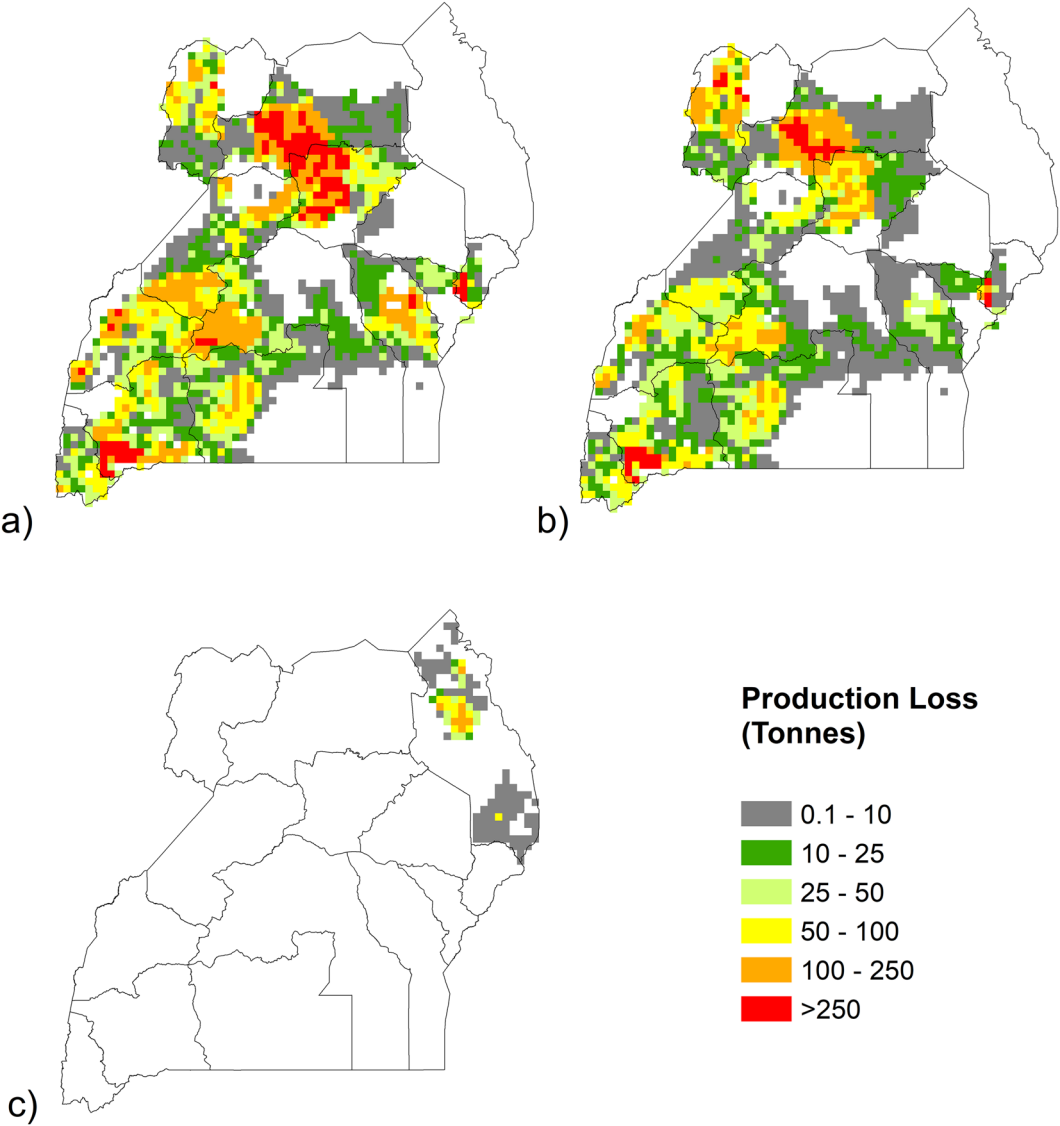
Total production loss (tonnes) due to ozone for Ugandan beans in 2015. For (**a**) growing season 1 (April–June); (**b**) season 2 (mid-Sep to mid-Dec) and (**c**) Karamoja sub-region (growing season July to Sep). Map based on 0.0833° × 0.0833° grid cell resolution production data, using the 2017 SPAM dataset. Blank cells show areas where there was no bean production in 2015. The maps in the figure were generated using ArcMap (version 10.6), https://www.arcgis.com.

The highest bean production losses due to ozone generally occurred in the areas of Uganda with high production. Production loss totals were also influenced by the levels of ozone flux and the split of production between the two seasons (Figure S4). Total estimated production losses due to ozone were higher in season 1 (114,355 tonnes) than in season 2 (70,137 tonnes) (Fig. 5, Table 2). In season 1, the highest losses were in the northern sub-regions of Acholi and Lango, and the western sub-regions of Ankole and Bunyoro. In season 2, the sub-regions with the highest production losses were Acholi and Ankole. For West Nile, while the estimates for potential yield loss were higher for season 1 than season 2 (due to higher levels of ozone flux), total production losses were higher in the second season, as the breakdown of production for this sub-region between the two seasons is 30:70. For the Lango sub-region, production losses were considerably lower in the second season, due partly to lower levels of potential yield loss (due to lower ozone flux), and also because production is higher in the first season than the second (69:31). Similarly for North Buganda in the central region, production losses are > 50% lower in the second season, due to slightly lower yield losses in season 2 and higher production in season 1 than in season 2 (61:39). Some areas with high production, for example, the Kigezi sub-region, show relatively low production loss which can be attributed to low levels of ozone flux.

**Table 2:** Production losses (tonnes) of beans in Uganda due to ozone, per sub-region for 2015.

Sub-region	Region	Production loss (tonnes)		
		Season 1	Season 2	Total
North Buganda	Central	10,235	5602	15,837
South Buganda	Central	5662	5365	11,027
Bukedi	Eastern	899	604	1503
Busoga	Eastern	6134	1409	7543
Elgon	Eastern	3785	1705	5490
Teso	Eastern	13	6	19
Acholi	Northern	18,496	15,556	34,052
Lango	Northern	20,773	7405	28,178
West Nile	Northern	5127	9300	14,427
Karamoja	Northern	2338	NA	2338
Ankole	Western	19,825	11,077	30,902
Bunyoro	Western	10,722	4385	15,107
Kigezi	Western	1853	1835	3688
Tooro	Western	8494	5888	14,383
Total		114,355	70,137	184,492

### Additional crop stresses

**Figure 6:**
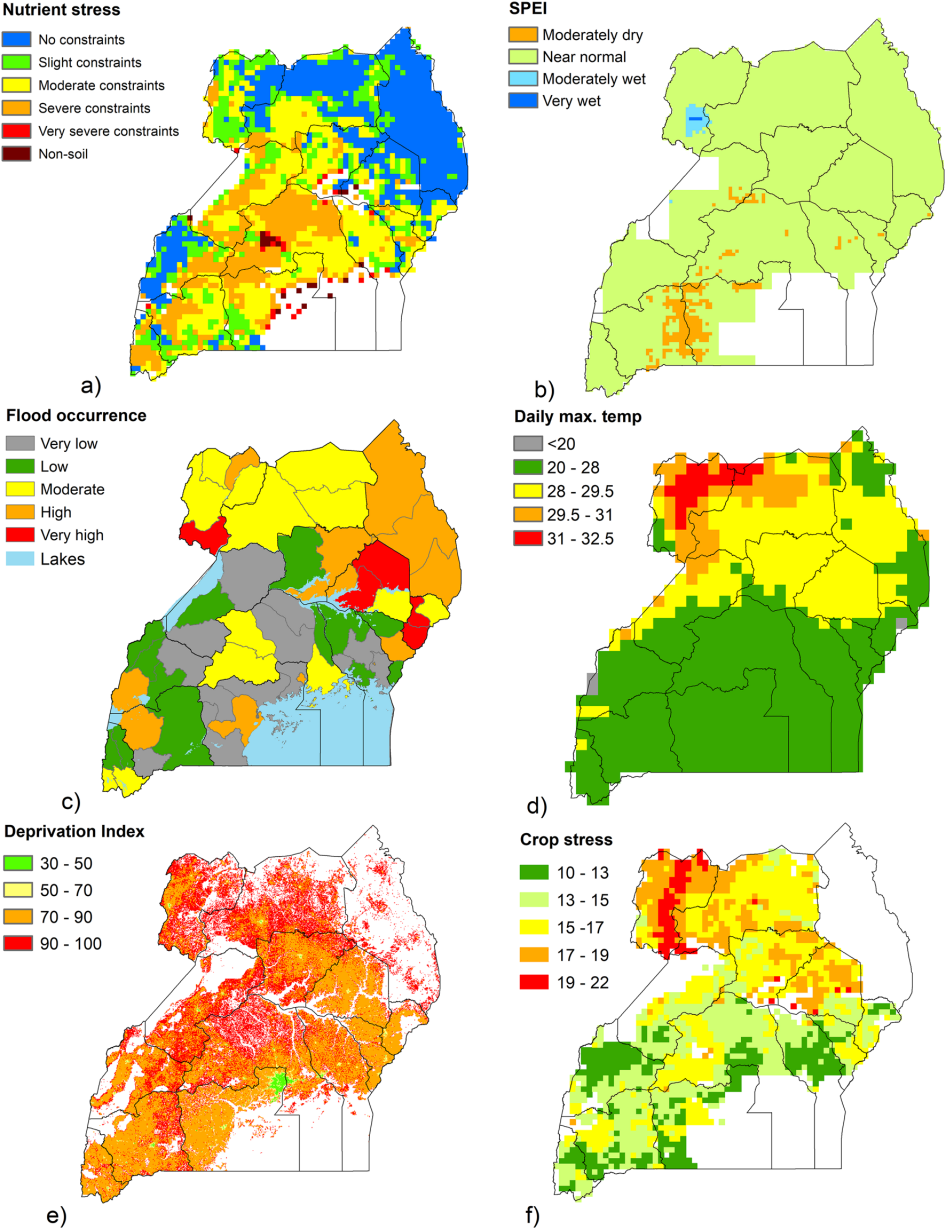
Spatial distribution of crop yield stresses in Uganda (0.0833° × 0.0833° resolution). For (**a**) Soil nutrient stress; (**b**) Standardized Precipitation Evapotranspiration Index (SPEI), 6-month accumulation, average value for period April to June 2015; (**c**) Flood occurrence (2007–2015); (**d**) Mean daily maximum temperature (May–June 2015); e) Relative Deprivation Index, 2010–2020; and (**f**) Crop stress score, combining all measures of stress, including ozone (as % yield loss), during the first common bean growing season. The maps in the figure were generated using ArcMap (version 10.6), https://www.arcgis.com.

Potential sources of crop stress in Uganda (for the first growing season), were found to vary spatially across the country (Fig. 6). Soil nutrient stress was generally higher in central and southern areas of the country, particularly in North Buganda (Fig. 6a). While 2015 was not a drought year for Uganda, the average SPEI values for the period April to June show some moderately dry areas in the south of the country (in Ankole and South Buganda), and also a wetter area in the north (West Nile sub-region) (Fig. 6b). The monthly SPEI values for 2015 (Fig. S6, Supplementary Information) provide further detail on dry and wet areas, with a band of moderately and severely dry conditions across the southern half of the country in May, and moderately to extremely wet conditions in northern areas, particularly the north-west, in June. Flood occurrence (for the period 2007 – 2015) varied across the country (Fig. 6c), tending to be highest in northern and north-eastern areas, with the highest risk districts including the Nebbi district in northern Uganda and the Katakwi, Soronko and Mbale districts in eastern Uganda. Daily max. temperatures (May–June 2015) were also highest in the north of the country (Fig. 6d), particularly in the West Nile sub-region, with values higher than optimum for common bean. The deprivation index level was generally high across the country, especially in rural areas (Fig. 6e). Lower values were seen in towns and cities, particularly around the capital city of Kampala.

When all stresses, including ozone, were combined to create a stress score (Fig. 6f), the highest stress levels were in the northern and eastern regions of Uganda particularly in the West Nile sub-region. This was due to a combination of moderate soil constraints, moderate to very wet conditions (SPEI), moderate to very high flood occurrence, very high daily max. temperatures, high deprivation levels and estimated ozone yield losses of > 22%. The stress score was also high in the Eastern sub-region of Teso, due to very high flood risk, high daily max. temperatures during the growing season, high deprivation and estimated ozone yield losses of 16–22% . The Karamoja sub-region hasn’t been included here as beans are grown at a different time of year, however this sub-region has its own issues with poverty, drought (in certain years), high flood risk and conflict, although ozone and soil nutrient stress are relatively low.

## Discussion

### Ozone impacts on bean farming in Uganda

Using crop production data, modelled ozone flux, and localised data on bean growing in Uganda, areas of the country at high risk of production losses due to ozone are highlighted. As the potential yield for beans in Uganda is considerably higher than actual yield^17^ investigating ozone impacts on bean yield could provide one way to address this yield gap.

The areas at greatest risk of production losses due to ozone were found to be in the northern sub-regions of Acholi and Lango, the western sub-regions of Ankole and Bunyoro (season 1), and Acholi and Ankole (season 2). Acholi and Lango are mostly self-sufficient in bean production, with some areas of minor deficit^16^. These losses could potentially be contributing to deficits in some areas, and as the bean farming system in Uganda is predominantly small-scale, individual households could be suffering detrimental effects due to ozone pollution. The highest bean surplus areas are in towns of North Buganda in the central region, and the western region of Uganda also has a minor surplus of bean production^18^. Bean losses due to ozone in these areas for example, could represent lost opportunities for profits from exporting beans to other areas of Uganda and/or neighbouring countries. The eastern sub-region of Karamoja has a deficit of bean production^18^. While production losses due to ozone are lower here, the average estimated yield loss is 11%, which could add to the problems already caused by drought and other factors in this sub-region.

### Additional stresses

There are areas of the country where multiple crop stresses coincide. In the eastern area with high stress scores (Teso sub-region), there is very little bean production, presumably because farming is unprofitable in this area. However, there are other areas with moderate to high stress scores where beans are being produced, for example West Nile and Acholi. The high levels of deprivation in the country may mean that it is difficult for farmers in rural areas to access improved bean cultivars and the majority of small-scale farmers practice low input farming. The sub-region of Karamoja has not been focused on in this study, however much of the sub-region is currently (as of Jan. 2024) under ‘Crisis’ in the Acute Food Insecurity classification of FEWSNET (https://fews.net/east-africa/uganda), due to climate-related impacts on crop production, endemic pests and diseases and a fragile security situation^62^. A greater awareness of the impact of ozone on bean production could allow farmers to address the yield gap in areas with high crop stress, using approaches that may not have been previously considered.

### Sources of variability

While this analysis uses modelling methods approved for use by the LRTAP Convention^49^ and data from the widely used EMEP model, there are some sources of uncertainty associated with the methodology.

There are potential differences between the bean production values from the spatial data (SPAM 2017) and the 2018 Ugandan agricultural survey dataset, when sub-regional totals are examined. This is to be expected, as both datasets are based on estimates, from different years, using differing methodologies (modelling and extrapolation vs. surveys of a subset of households respectively). The total estimated production value for beans in Uganda in 2018, from the national survey, was ~ 727,000 tonnes and the SPAM 2017 estimated total was ~ 1 Million tonnes. For further comparison, using data from the Food and Agriculture Organisation of the United Nations (FAOSTAT; https://www.fao.org/faostat/en/), the national bean production total for Uganda in 2015 was 1,079,943 tonnes, in 2017 was 1,012,406 tonnes and in 2018 was 940,323 tonnes. There is some uncertainty in all estimates (and therefore the resulting totals for production losses due to ozone), however the FAO totals suggest that the SPAM dataset is not greatly overestimating production and does reflect production values per sub-region for the time period investigated.

At the time of running the EMEP model for this study, the most up-date-emissions data available were for 2015, while the SPAM dataset was only available for 2010. The 2017 SPAM dataset was subsequently released, therefore as this was the closest time period to the emissions data, this dataset was used to map bean production in Uganda with conversion factors for each region used to convert the 2017 production data to 2015 values per grid cell, based on how production per region had changed between the two national agricultural surveys (2008/09 and 2018). The survey data were used as these are the only datasets providing Ugandan crop production data at a regional scale. While this method uses data on Ugandan bean production to inform how production may have changed, it does not account for smaller scale changes within regions, and fluctuations between 2009–2018. The SPAM production data were at a resolution of 0.083° × 0.083°, while the EMEP data were 0.3° × 0.3°. Therefore, some further variability could arise from using datasets with different resolutions. The use of finer scale ozone data would provide further information on localised patterns in ozone concentration/uptake. The EMEP model is continuously improved and updated, so this should be possible for future studies.

For the current study, the EMEP-WRF model was run for 1 year (2015), due to computational constraints. Ozone concentrations can be expected to vary between consecutive years^63^, primarily due to variations in weather and, longer term, due to emissions of precursors. Future modelling studies for the African region should be carried out with further years of data, preferably using an average across years, to allow for annual variation in ozone levels. The EMEP modelled data for 2015 in the current study showed the highest ozone concentrations in December (~ 62 ppb) in south-eastern and also central-western areas of Uganda. Ozone concentration in a location does vary between years, due to changes in emissions and also, shorter term, due to weather conditions, so it is not surprising that measured values were slightly higher in 2018/19^7^. The few monitoring studies that have been done in Uganda show that mean ozone concentrations can be high at certain times of the year, particularly in rural locations. As surface ozone is predicted to increase in future decades, for example in equatorial Africa, ozone impacts on crops in Uganda could be greater than estimated in the current study in future years so it is important to highlight the potential damage this pollutant can cause.

When calculating ozone flux for this study, a 90-day accumulation period centred around the ozone sensitive period between anthesis and the end of grain fill was designated for the two growing seasons in Uganda. These periods were chosen to reflect the key growing times across the country, however growing periods (particularly for the second growing season) can vary slightly depending on the area and variety of bean. Also, the harvest time for the Karamoja growing season is broad (August to November). According to the EMEP data, Ugandan ozone concentrations don’t vary greatly between month, however there is some fluctuation. Therefore, there may be some variation in estimates of ozone impact, depending on the 90-day period selected to calculate ozone flux.

The bean flux-effect relationship used here includes data from 7 bean cultivars from around the world, including Pinto, Mbombo and Black Turtle (grown in Kenya) and Orca (grown in South Africa) (See Supplementary Information, Sect. 4). The relationship could be further strengthened by including more cultivars, as different bean cultivars can vary in sensitivity to ozone^9^. For the case of Uganda, it would also be useful to test the ozone sensitivity of popular cultivars that are grown widely, including those developed by NARO. This would improve estimates of ozone impacts on bean growing in Uganda, and farmers could be informed on which cultivars were more ozone tolerant and could lead to a potential increase in yield.

Ozone impacts on crop production for Sub-Saharan Africa have previously been investigated on a large scale^13^ and included results for Uganda. While the study used the same modelled O_3_ dataset from the EMEP WRF model, there were some differences in the results from this large-scale investigation, compared to the current study. Results presented here give an improved prediction of the magnitude of ozone impacts on production in Uganda, and how this varies with growing season. Growing season dates have been refined and a separate season for Karamoja is used. Estimated yield losses for Karamoja for the July to September season were generally lower than for the season used by the previous study^13^ (Oct-Dec; average difference of 1.24%, +-1.33 sd; range from − 1 to 3%). For production data, the current study used the most up-to-date SPAM dataset (2017), and estimates were considerably higher (compared to the 2010 dataset). Ugandan agricultural survey data on production (per region) and production of beans per growing season (per sub-region) were used, allowing more informed estimates of ozone impacts. Overall, total bean production loss estimates due to ozone for Uganda were higher in the current study (184,000 tonnes), compared to those estimated by the larger scale study^13^ (86,000 tonnes), and revealed the difference in production losses between the two main growing seasons. Results are provided at a more detailed level than the previous study, allowing discussion of implications of ozone impacts for different areas of the country.

The datasets on additional crop stresses each have their own sources of variability^2,51,57,58,61^, and time periods vary depending on the data source. Drought levels and dryness vary spatially between years, which can be seen in the SPEI dataset (which provides monthly data for a 35-year period). Therefore, areas where the different stresses coincide will vary with year. The flood occurrence data are annual data and don’t specify when in the year the flood took place, however as floods generally occur in the two Ugandan rainy seasons^53^, which is also when crops are grown, this is a good indication of risk to crop yield. The stress score calculated is a useful indicator on where crop stress may be particularly high, however it does not account for possible interactions between stresses. For example, there can be interactions between drought and soil stresses such as fertility and toxicities^64^, and a warming climate is predicted to lead to population growth and increased metabolic rates of insect pests^65^ (another important crop yield constraint). Water-logged soil can also lead to emergence of diseases and pest damage^31^.

### Mitigation options

There are a number of potential options for reducing ozone impacts on crops. The most effective method would be to lower emission levels of ozone precursors, and global efforts are being made towards this goal.In the Southern African region, the most effective emission control strategy to reduce ozone levels should be CO and VOC reduction, mainly associated with household combustion and regional open biomass burning^6^. Over 90% of households in Uganda use solid biomass fuel (charcoal) for cooking^66^. Uganda joined the Climate and Clean Air Coalition (CCAC) in 2021, and there are aims to gather more air pollution data in the country. It would be useful to collect more data on ozone concentrations, particularly in rural areas, during the growing seasons. As hemispheric transport of ozone can occur, the pollutant (and its precursors) can also travel long distances from their sources. Therefore, it is not enough to only consider emissions in the local area, co-operation between countries is required.

There are also other short-term options that can be considered to reduce ozone impacts, for example following particular crop management strategies. With more information on ozone levels in Uganda at different times of the year, it could be possible to time planting to coincide with lower levels of ozone. Currently, levels of crop irrigation in Uganda are low, however the Ugandan government introduced an irrigation masterplan^67^ in 2017, aiming to increase the irrigated crop area in the country to tackle the effects of climate change. While increasing irrigation can lead to higher levels of ozone uptake by crops, altering the timing and level of irrigation can impact how much ozone is taken into the plants. Reduced irrigation in Kenyan wheat stimulated grain weight and harvest index, which compensated for ozone induced reductions in well-watered plants^68^.

The International Centre for Tropical Agriculture (CIAT) and its partners, including NARO in Uganda, have developed a range of bean varieties with useful adaptations, including fast growing varieties and those tolerant to particular pests/diseases^69^. As different cultivars of the same crop show varying sensitivity to ozone, there is also scope for breeding ozone tolerant cultivars. It would, however, be important to balance ozone tolerance with other favourable characteristics.

In terms of mitigating other crop stresses, most farmers in Uganda use low-input practices due to resource constraints^16^. The primary methods for managing soil fertility include transfer of plant materials from non-cropped areas to arable land, biological nitrogen fixation through legumes and using manure^32^. Sustainable farming practices, such as conservation tillage, can improve soil water storage and increase the yield of Ugandan beans^70^. In areas subjected to frequent water-logging of bean crops, Ugandan farmers employ ‘indigenous coping mechanisms’, including planting the crops on ridges and construction of drainage channels^31^. The provision of weather and climate information could help at-risk communities cope with extreme events such as flooding and farmers do have some strategies available, for example planting early-maturing, water-tolerant crop varieties, however these can be difficult for farmers to obtain^71^. Using accessible techniques such as adding organic fertilizer and maize-legume intercropping could also help to reduce crop sensitivity to high temperatures^72^. As agricultural productivity in Uganda remains below its potential, changing practices to mitigate for losses due to ozone pollution, could be another way to reduce the yield gap for bean production and help to improve economic returns for Ugandan farmers. However, more stringent policies to reduce ozone pollution in the region are also required, as some local growers may not have the resources to mitigate impacts.

## Conclusions

This study highlights areas at risk of yield and production losses due to ozone for an important food crop in an African country highly dependent on agriculture. Results demonstrate how the use of detailed, local data can improve predictions on the magnitude of production losses due to ozone and how this varies during the seasons. Results also highlight that the areas of greatest risk of ozone reductions in bean yield can coincide with other stresses, which puts additional pressure on a population already struggling to meet dietary needs. Opportunities exist to improve the bean industry in Uganda, including an established research infrastructure to promote bean production and the availability of a wide range of improved bean varieties^17^. The methodology used here can be applied widely, for countries with ozone data (preferably modelled and measured to allow for model validation), crop production data and spatial data on other stresses. Ozone flux-effect response functions are available for crops, some tree species and semi-natural vegetation^49^. As agricultural productivity remains below its potential in many areas of the world, addressing the yield gap due to ozone could provide benefits for farmers, particularly as changes in climate and continuing rises in emissions are predicted to lead to further increases in ozone in future years^8,9^.

### Supplementary Information

Supplementary Information.

## Data Availability

The datasets used and/or analysed during the current study are available from the corresponding author on reasonable request. Due to requirements of the NERC funding body, if requested, data would be made available via the Environmental Information Data Centre (EIDC), where data could be downloaded.
