# Supplementary material for: Ozone pollution contributes to the yield gap for beans in Uganda, East Africa, and is co-located with other agricultural stresses
Source: Sci Rep. 2024 Apr 5;14:8026. doi: 10.1038/s41598-024-58144-1 (PMC10997645; doi:10.1038/s41598-024-58144-1)
Supplement: Supplementary file 1 — Supplementary Information. [file 41598_2024_58144_MOESM1_ESM.pdf]

Supplementary Information

Sharps *et al.* 2024. Ozone pollution contributes to the yield gap for beans in Uganda and is co-located with other agricultural stresses.

Sharps, K.[1*], Foster, J.[1], Vieno, M.[2], Beck, R.[2] & Hayes, F. [1]

[1]UK Centre for Ecology & Hydrology, Environment Centre Wales, Deiniol Road, Bangor, Gwynedd, LL57 2UW, UK.

[2]UK Centre for Ecology & Hydrology, Bush Estate, Penicuik, Midlothian, EH26 0QB, UK.

**Corresponding author*: Dr Katrina Sharps, katshar@ceh.ac.uk; Phone: 00 44 1248 374500.

1. EMEP model parameterisations

Table S1: Input parameterisation for land-cover definitions for EMEP-WRF Africa.

CROP	#Name	code	type	PFT	hveg	Alb	eNH4	SGS50	DSGS	EGS50	DEGS	LAImin	LAImax	SLAIlen	ELAIlen	BiomasD	Eiso	Emtl	Emtp	Eiso-m2	Emt-m2
	#				m	(%)	day	days/d	day	days/d	day	m2/m2	m2/m2	days	days	g/m2	ug/g/h	ug/g/h	ug/g/h		
Bean	IAM_AfrBe_NonIrrig	IAM_AfricaBean_NonIrrig	ECR	NOLPJ	1	20	0					4	4	1	1	0	0	0	0	0	0

Where:

IAM_AfricaBean_NonIrrig = POD_3IAM for non-irrigated African beans

<u>User notes</u>:
Start and end days of the growing season were not needed for these model runs as daily values of POD_3IAM were used for this exercise.

hveg = Height of vegetation, Alb = Albedo, ENH4 = Flag for possible Nhx fluxes
LAI = Leaf Area Index
LAImax - give as -1 if bulk resistance
SLAIlen = days from LAImin to LAImax at start of season
ELAIlen = days from LAImax to LAImin at end of season
(Set SLAIlen and ELAIlen to 1 for vegetation with constant LAI)
BVOC biomass loosely based upon Simpson et al., (1999)
BVOC data only used outside Europe as defaults
'type' - used in deposition system, e.g, to define areas where N-dep to coniferous forest is calculated
ECF - conif forest; EDF - decid forest; SNL - seminnatural; W - Water

Table S2: Input parameterisation for DO_3SE within EMEP-WRF Africa

For explanation of parameters see EMEP user manual (http://emep.int/mscw/index_mscw.html)

CROP	# Code	Landcover	gmax	fmin	f_phen	#	#	#	#	#	Astart	Aend	flight	ftemp	#	#	Surface	Res.	fVDP	#	VPD	fSWP	#	rootd	Lw,,,,
	#Code	LU	#	#	fac	fac	fac	fac	len	len	(rel-SGS)	(rel_EGS)	#	min	opt	max	RgsS	RgsO	max	min	Crit	SWPmax	PWP	m	m,,,,
	#	#	#	#	a	b	c	d	e	f	days	days	#	#	#	#	#	#	#	#	#	#	#	#	#
Bean	IAM_AfrBe_NonIrrig	IAM_AfricaBean_NonIrrig	415	0.01	1	1	1	1	1	1	0	0	0.02	15	29	43	150	200	2.8	4.5	8	-9.99	-99.9	0.6	0.03,,,,

Where:

IAM_AfricaBean_NonIrrig = POD_3IAM for non-irrigated African beans

2. EMEP model validation

When working with data from chemical transport models, it is important to ensure that modelled values are representing the atmosphere as adequately as possible, therefore (where possible) outputs are compared with measurements in a validation process. There is no established network of monitoring sites in Uganda as there are in some other African countries, for example South Africa. There have been a small number of studies looking at levels of air pollution in Uganda using low-cost sensing and measurement technologies.

For example, Kirenga et al. (2015) measured ambient ozone concentration in two of the largest cities in central Uganda (Kampala and Jinja) using real time monitors, for the period 30th June to 27th July 2014. Ozone concentrations were found to be low, below the WHO guideline levels of 50ppb eight-hour mean, for example, the mean one-hour O3 concentration at the central monitoring site in Kampala measured over a period of seven days was 11.4 µg/m3 (5.8ppb). However, despite the authors placing monitoring devices in different land use types across the cities (commercial, industrial, residential), all the monitoring sites were in urban areas. Ozone concentrations are often considerably lower at urban sites compared to rural because there is a titration effect, with ozone being degraded by the compounds (NOx) by which it is also formed. This degradation occurs more often in cities than in rural areas, because there is more NO in cities. Therefore, to get a representative idea of ozone concentration across the country, monitoring sites need to be placed in both urban and rural areas.

More recently, Okure et al. (2022) deployed 120 diffusion tubes in Dec 2018 – Feb 2019 in selected urban centers in three of the four macro-regions in Uganda. Ozone concentrations varied spatially and with time, and two-monthly means ranged from 32.59ppb to 82.69ppb, with the highest values in South-Eastern Uganda, and values of >70ppb close to Kampala, and 50-60ppb at sites in central and Western Uganda. As expected, smaller urban centres and rural locations experienced higher O_3 concentrations. The modelled data for 2015 in the current study showed the highest ozone concentration values in December (~62ppb) in South-Eastern and also central-Western areas of Uganda. Ozone concentration in a location does vary between years, due to changes in emissions and also shorter term due to weather conditions, so it is not surprising if values vary between the measured values in 2018/19 and the modelled values in 2015.

The EMEP model is used widely to estimate levels of air pollutants around the world. Modelled values have been validated using a variety of exercises. For example, Mills et al. (2018a), used the EMEP model to provide ozone concentration and flux data for the period 2010-12 to estimate the global impact of ozone on wheat production. The approach used to test the model's validity is explained in detail by the authors in the Supplementary Information of the paper. Over the course of a year, the EMEP model was found to capture the spatial and temporal variations in daily maximum hourly mean ozone concentration (Dmax) across the regions tested (Ireland, Germany, USA, Canada, Argentina, Japan) well, including the seasons with higher concentrations and longer ozone episodes. A very good correlation between the modelled and observed values (r^2 ranges from .88 to .95) was also found for model estimated Dmax and M7 (7-hour mean) for Global Atmosphere Watch (GAW) network sites in continents across the world, including some sites in Africa.

Similarly, Pandey et al. (2023) used the EMEP model to investigate ozone impacts on wheat production in India (for the period 2008-12). Again, a good overall performance of the EMEP model was found for most global sites from the GAW network. Unfortunately, there are no rural sites from India in the GAW network, however a good-quality dataset for model evaluation was available for one semi-urban site, Mohali (northern India). The model was found to generally overpredict the daily mean (Dmean) ozone levels (ca. 58% over January to March, the POD_3IAM accumulation period for most sites). However, daytime ozone (M7 and the 12-h daytime metric M12) was captured better than night-time

concentrations. This implies greater certainty of modelled daytime ozone concentrations which are used to derive the POD_3IAM values, but the lack of rural monitoring data meant that it wasn't possible to quantify the uncertainty for rural sites any further.

For Africa, unfortunately there are not many measured datasets available to validate the EMEP model estimates for ozone concentrations/flux. There were three African sites with measured ozone data for 2015 available in the GAW and Tropospheric Ozone Assessment Report (TOAR) observation portals. The monitoring sites were on mountain tops (Kenya and Canary Islands) and in a coastal location near Cape Town, South Africa (Fig. S1). The 2015 data for South Africa show that while there were some fluctuations, the general temporal pattern of ozone concentrations was similar between the modelled and observed data. For the purposes of this current study, it would be preferable to have monitored data from rural locations in Uganda/neighbouring countries.

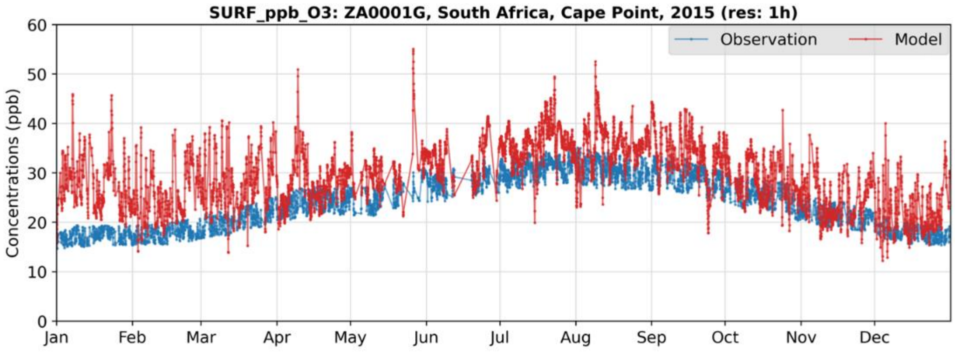

Figure S1. Hourly surface ozone concentrations measured for the year 2015 at Cape Point, South Africa, and modelled ozone concentrations from the EMEP-WRF Africa model.

In Asia, Europe and North America, the primary source of ozone precursors is from vehicles and industry however in Africa, additional sources include household combustion and regional open biomass burning. Combustion from households is very difficult to calculate and for this work emissions estimates that have been compiled globally were used. Although very important, new emissions estimates have not been created as such activity was beyond the scope of the study. For the investigation of ozone impacts in Uganda, we used what was accepted as state of the art at the time when the calculations were performed. The emissions used in the model were derived from the ECLIPSE (Evaluating the Climate and Air Quality Impacts of Short-Lived Pollutants) model (2015, version 6a). For further information on the ECLIPSE model, see Stohl et al. (2015).

The weather data used to drive the EMEP model is from the Weather Research and Forecast (WRF) model. The outputs from this model (including wind speed, precipitation, temperature, relative humidity) have been validated in a number of studies using locations around the world, for example in Greece (Emmanouil et al., 2021) and Central India (Kadaverugu et al., 2021). In Africa, Mazzeo et al. (2022) tested WRF model estimates of temperature, relative humidity, wind speed and direction (while running with the Chimere chemical transport model, predicting $PM_{2.5}$ levels) in Addis Ababa in Ethiopia, Nairobi in Kenya, and Kampala in Uganda. Results from the validation of the meteorological simulations performed over East African using WRF showed that the model is, on average, able to reproduce all four of the considered variables, providing values close to the observed data, with variable agreement between the three cities. When the WRF model is run with EMEP, the WRF model output is evaluated for surface temperature, relative humidity, and wind speed/direction using the National Oceanic and Atmospheric Administration (NOAA) dataset.

Overall, it would be useful to perform further validation tests with measured ozone data from Eastern Africa, however this is currently not possible. The EMEP model performs well on a global scale (as does the WRF model). The results of this study investigating ozone impacts on crop yield in Uganda suggest that there is potential for considerable yield losses in common bean crops, which is backed up by the ambient concentrations of ozone measured in rural areas in the few studies available for Uganda. Further work, including carrying out further ozone monitoring at rural sites around the country (at regular intervals if possible) is required to confirm the estimates presented here.

3. Gathering local data on bean production in Uganda

Table S3: Seasonal crop calendar for a typical year in Uganda. Source: FEWSNET (https://fews.net/east-africa/uganda)

Month	Bimodal season	Unimodal season
November	Weeding	Main harvest
December	Second season harvest	
January		
February	Land preparation	
March		
April	Planting	Land preparation
May		
June	Weeding	Weeding
July	First season harvest	
August		
September	Land preparation	Main harvest
October		

Table S4 – regional conversion factors for beans (converting 2017 production values to 2015 values)

Region	Prod 2008/9	Prod 2018	Difference	Diff/yr	Annual CF	2 yr CF
Northern	251,221	74,117	-177,104	-18643	-0.0742	-0.15
Eastern	98,834	95,032	-3,802	-400	-0.0040	-0.01
Western	411,945	342,594	-69,351	-7300	-0.0177	-0.04
Central	167,276	215,909	48,633	5119	0.0306	0.06

Regional production data for 2008/9 are from the UCA 2008/2009 country-wide agricultural survey (UBOS 2010), while data for 2018 are from the AAS agricultural survey (UBOS, 2020).

For each region, the following equation was used to convert 2017 SPAM production data (IFPRI, 2020) to an estimate of 2015 bean production per 0.0833 grid cell:

Production in 2017 - (Production in 2017 * 2yr CF)

4. Development of ozone flux-response relationship for bean

Experimental work was recently carried out to develop the first ozone flux-based relationship for common bean at the UKCEH solardome site in Bangor, North Wales, UK (Hayes et al., 2019). The aim of this experiment was to estimate ozone impacts on crops, including common bean, in Africa, particularly East Africa as bean production is high in this area. The final flux relationship included bean varieties grown in tropical countries, such as Mexico/South America (Tiger beans), India (Rajama) and African countries such as Kenya (Pinto, Mbombo, Turtle) and South Africa (Orca). No Ugandan beans were grown during this experiment as farmers use a large number of cultivars, some of which are very localised. However, Cannellini beans are white beans, which are grown in some areas of Uganda, and three of the seven cultivars are grown in neighbouring Kenya. We included all cultivars in the bean flux-response relationship in this study for Uganda as it is important to try and account for the possible differences in sensitivity/ozone response between cultivars, allowing for a more robust prediction of impact on yield.

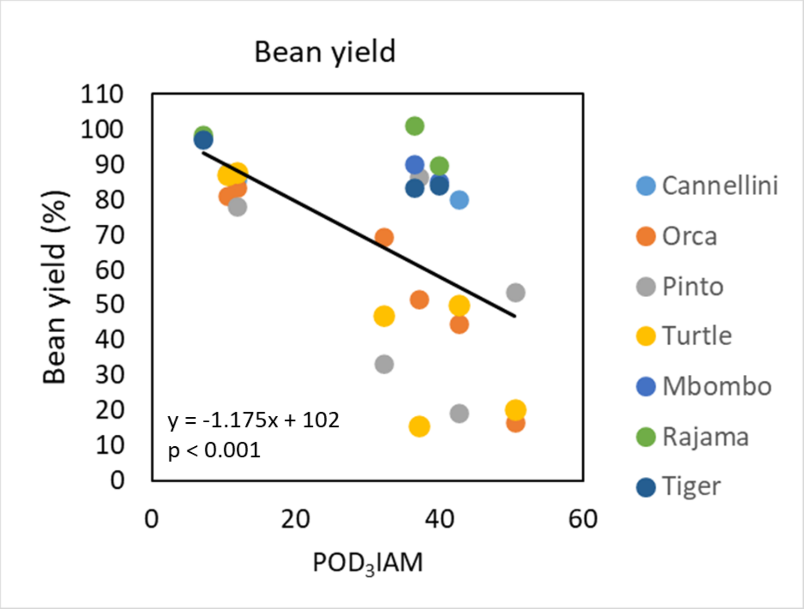

Figure S2. Relationship between the percentage bean yield and stomatal ozone flux (POD_3IAM) for common bean. The relationship is based on experimental data collected in the UKCEH Bangor solardomes for seven bean cultivars during the growing seasons of 2017-2019. The regression of yield with POD_3IAM followed the approach used by Fuhrer (1994). For each bean cultivar, yield was calculated at zero POD_3IAM by obtaining the intercept value from the regression model. This was then used as the reference level for no effect when calculating relative yield ('Bean yield (%)' in this figure).

The ICP Vegetation, an international research programme investigating air pollution impacts on vegetation (including crops) has been building a crop sensitivity to ozone database, which is updated regularly with published experimental data on ozone impacts on crops. For beans, the majority of the datasets use 7-hour mean (M7) ozone concentration data. In the Uganda study, we use ozone flux, which has been shown to perform better than concentration-based models, providing improved predictions of the distribution of ozone damage. The LRTAP (Convention on Long-range Transboundary Air Pollution) Manual for Modelling and Mapping (Chpt.3) provides flux-based response relationships for a number of crops, (wheat, tomato and potato), however this is the first time that an ozone flux-response relationship has been developed for beans.

https://icpvegetation.ceh.ac.uk/sites/default/files/FinalnewChapter3v4Oct2017_000.pdf

When the M7 bean data in the ozone sensitivity database are combined (Fig. S2), statistical analysis shows that the newer data (orange points) collected in the UKCEH Bangor solardomes (Hayes et al., 2019) and used for the ozone flux-relationship fit well with those already in the database, with the ozone sensitivity of these cultivars typical compared to the other legumes. Statistical analysis showed that while there was a strong relationship between 7-hour mean ozone and relative yield ($p < 0.0001$), there was no difference between the slopes for the two datasets ($p = 0.46$).

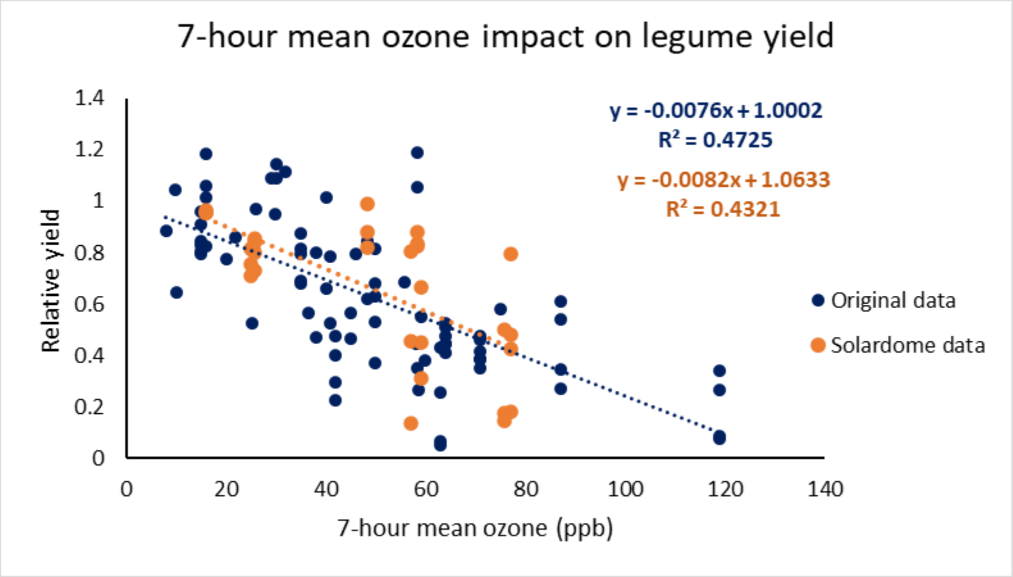

Figure S3. Legume (peas, beans and peanuts) data in the ozone sensitivity crop database from the ICP Vegetation spanning studies from 1988 to 2014, from locations in Europe, USA, Pakistan, India) using the 7-hour mean ozone metric (dark blue points). Data from the 2017-19 UKCEH solardome experiments using the 7 cultivars used in the Uganda study (including those growing in Africa) has also been included (orange points).

5. Estimating crop stress impacts on bean in Uganda (incl. ozone)

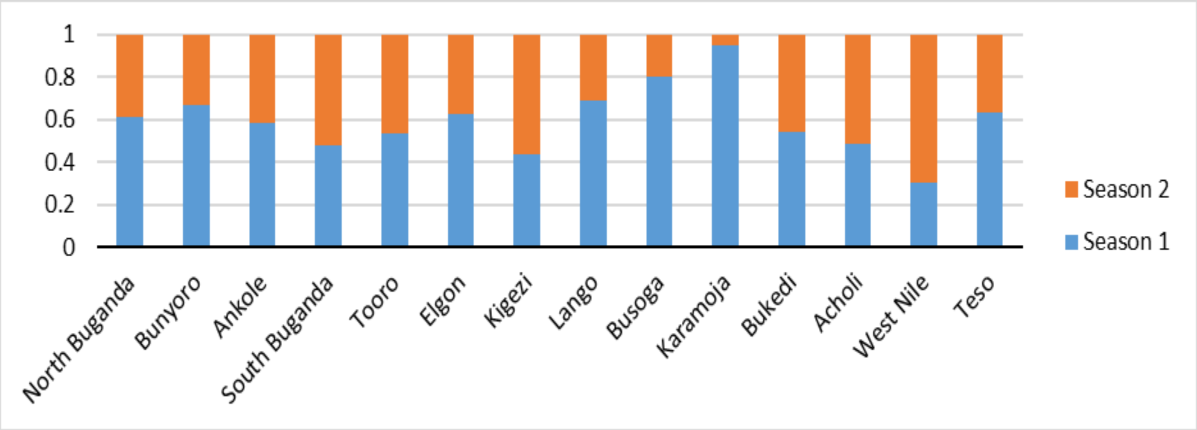

Figure S4: Seasonal production proportions for beans produced in Uganda in 2018. The data are taken from the 2018 Ugandan agricultural survey (UBOS, 2020), and are ordered from highest total annual production (North Buganda) to lowest (Teso).

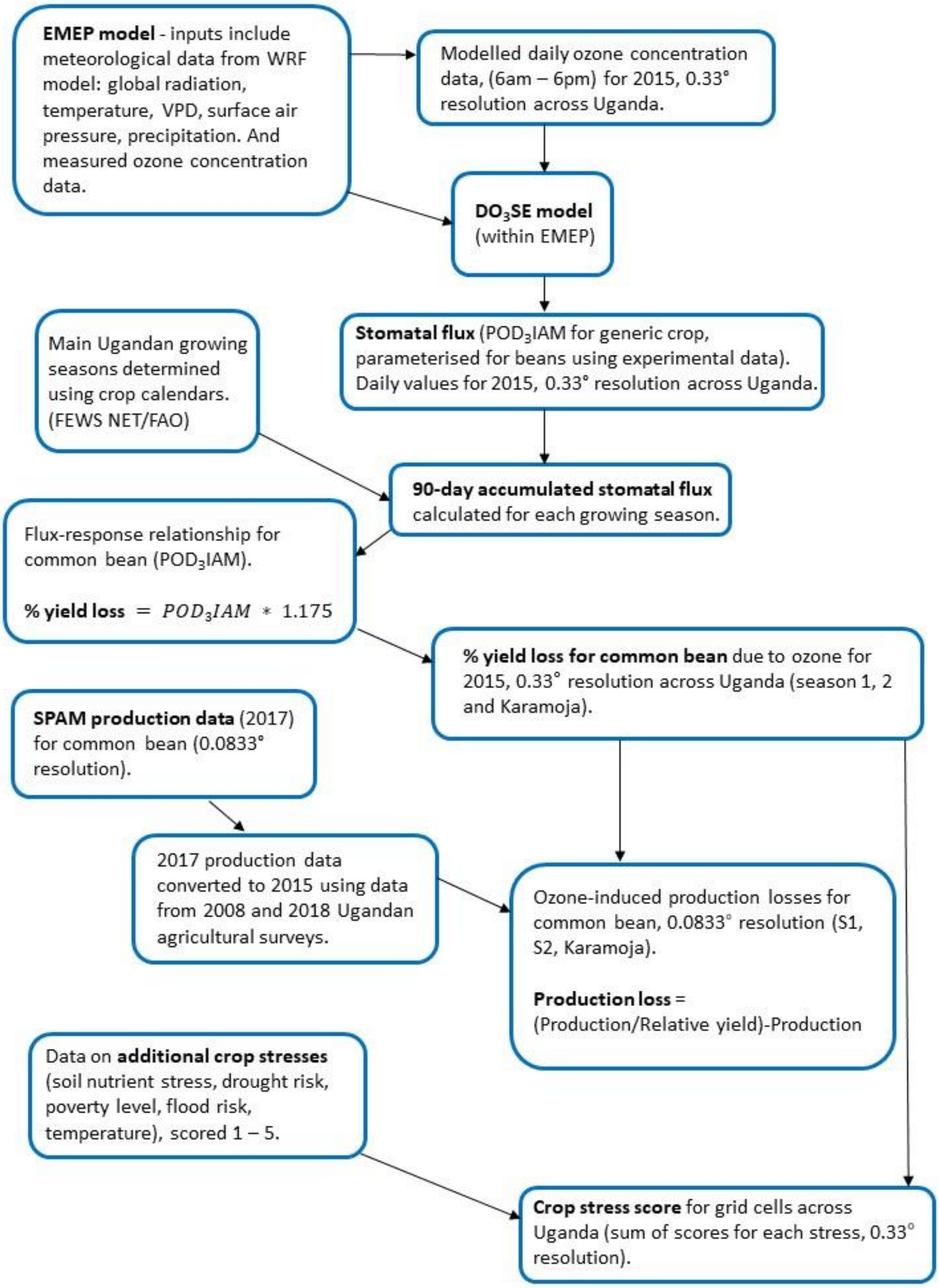

Figure S5. Flow chart of the methodology used in this study to estimate impacts of ozone on yield loss and production of common beans in Uganda. The EMEP model is the European Monitoring and Evaluation Programme chemical transport model (Simpson et al., 2012), which uses meteorological data from the Weather Research and Forecast (WRF) model. Within the EMEP model, the Deposition of O_3 for Stomatal Exchange (DO$_3$SE) model (Emberson et al., 2000) calculates ozone flux, using environmental data, including light, temperature and Vapour Pressure Deficit. Crop calendars were use from the Famine Early Warning Systems Network (FEWS NET) and the Food and Agriculture Organisation of the United Nations (FAO). The eastern sub-region of Karamoja has a unimodal growing season and was therefore included in the results separately from the main first and second growing seasons across the rest of the country.

Table S5. Categories of crop stress score for ozone, soil nutrient, deprivation, drought, temperature and flood occurrence. HWSD is the Harmonised World Soil Database and SPEI is the Standardised Precipitation-Evapotranspiration Index.

Stress	Attribute	Years of data	Yield constraint score				
			1	2	3	4	5
Ozone	% Yield Loss	2015	0 to 5	5 to 10	10 to 25	25 to 40	>40
Soil nutrients	Retention	HWSD data, 2009	None or slight	Slight to moderate	Slight to severe	Moderate to severe	Moderate to very severe
	Availability	Downloaded 2017.	None	Slight to moderate	Moderate to severe	Severe	Severe to very severe
	Overall		None	Slight	Moderate	Severe	Very severe
Drought	SPEI	2015 (6-month accumulation)	Near normal	Moderately wet Moderately dry	Very wet Severely dry	Extremely wet Extremely dry	
Flood Occurrence	No. of years with floods	2007 - 2015	0	1 to 2	3 to 4	5 to 6	7
Temperature	Daily maximum (mean)	2015 (May and June)	20-28°C	11-20°C	28-29.5°C	29.5-31°C	31-32.5°C
Level of deprivation	Deprivation Index (0 – 100)	Input datasets range from 2010 to 2020	0 to 25	26 to 50	51 to 70	71 to 90	91 to 100

Note: Soil nutrient data are from the Global Agro-Ecological Zones (GAEZ) data portal, derived from combinations of soil attributes, using data in the Harmonized World Soil Database (HWSD, v. 1.1, FAO/IIASA/ISRIC/ISS CAS/JRC 2009). The scoring system for soil nutrient stress (and % yield loss due to ozone) follow Mills et al. (2018b). The Deprivation Index is the Global Gridded Relative Deprivation Index (GRDI; v1), from NASA's Socioeconomic Data and Applications Centre (SEDAC) (CIESIN 2022). The SPEI data values have been computed for Africa at 5km resolution, monthly for the period 1981 to 2016 (Peng et al., 2020). Temperature data is from WorldClim (https://www.worldclim.org/), CRU-TS 4.06 (Harris et al., 2020) downscaled with WorldClim 2.1 (Fick and Hijmans, 2017). Flood occurrence data are from Mitheu et al., 2023 - flood impact data were collated from four different data repositories from 2007 to 2015, and the number of years out of the 9 years considered when floods occurred were summed for each district in Uganda.

Table S6: Estimated bean production per sub-region in Uganda, 2015, in tonnes (t), using 2017 SPAM data (IFPRI, 2020). The breakdown for each growing season (S1 and S2) is also given, per sub-region.

Sub-region	Total 2015 production (t)	S1 production (t)	S2 production (t)
Ankole	257,215	150,880	106,335
Acholi	134,218	65,604	68,614
Lango	114,840	78,840	36,000
Tooro	106,946	57,003	49,943
North Buganda	104,715	64,091	40,624
Bunyoro	75,742	50,592	25,150
South Buganda	72,371	34,519	37,852
West Nile	54,082	16,455	37,627
Kigezi	49,313	21,377	27,936
Busoga	39,754	31,804	7,950
Elgon	31,924	19,999	11,925
Karamoja	21,455	20,312	1,143
Bukedi	7,849	4,260	3,589
Teso	77	49	28
TOTALS	1,070,501	615,785	454,716

Table S7. Production totals for beans in Uganda, in tonnes, for 2008/9, 2015, 2017 and 2018. The values for 2017 have been taken from the Spatial Production Allocation Model (SPAM) dataset, and those for 2015 have been estimated using the 2017 SPAM dataset and conversion factors per region, based on regional data for production from the 2008/9 and 2018 country-wide agricultural surveys. Survey totals for 2008/9 and 2018 are also given for comparison. Note, the 2008/9 survey data did not provide data at the sub-regional level. The FAOSTAT (https://www.fao.org/faostat/en/) total production values for Ugandan beans are also shown.

Sub-region	Region	2008/9 survey (t)	Estimate of 2015	Totals	Spam 2017	Totals	AAS 2018 survey	Totals
North Buganda	Central		104715		111280		142433	
South Buganda	Central	167276	72371	177086	77089	188370	73476	215909
Busoga	Eastern		39754		39434		20373	
Elgon	Eastern		31924		31668		56701	
Bukedi	Eastern		7849		7195		14945	
Teso	Eastern	98834	77.12	79604	76.5	78374	3014	95033
Acholi	Northern		134218		116872		10168	
Lango	Northern		114840		100000		39728	
West Nile	Northern		54082		46010		9002	
Karamoja	Northern	251221	21455	324594	18682	281564	15218	74116
Ankole	Western		257215		248411		91280	
Tooro	Western		106946		103286		69551	
Bunyoro	Western		75742		73149		134364	
Kigezi	Western	411945	49313	489216	47625	472471	47399	342594
**Uganda total**		929,276		1,070,501		1,020,779		727,652
**FAO**		925,000		1,079,943		1,012,406		940,323

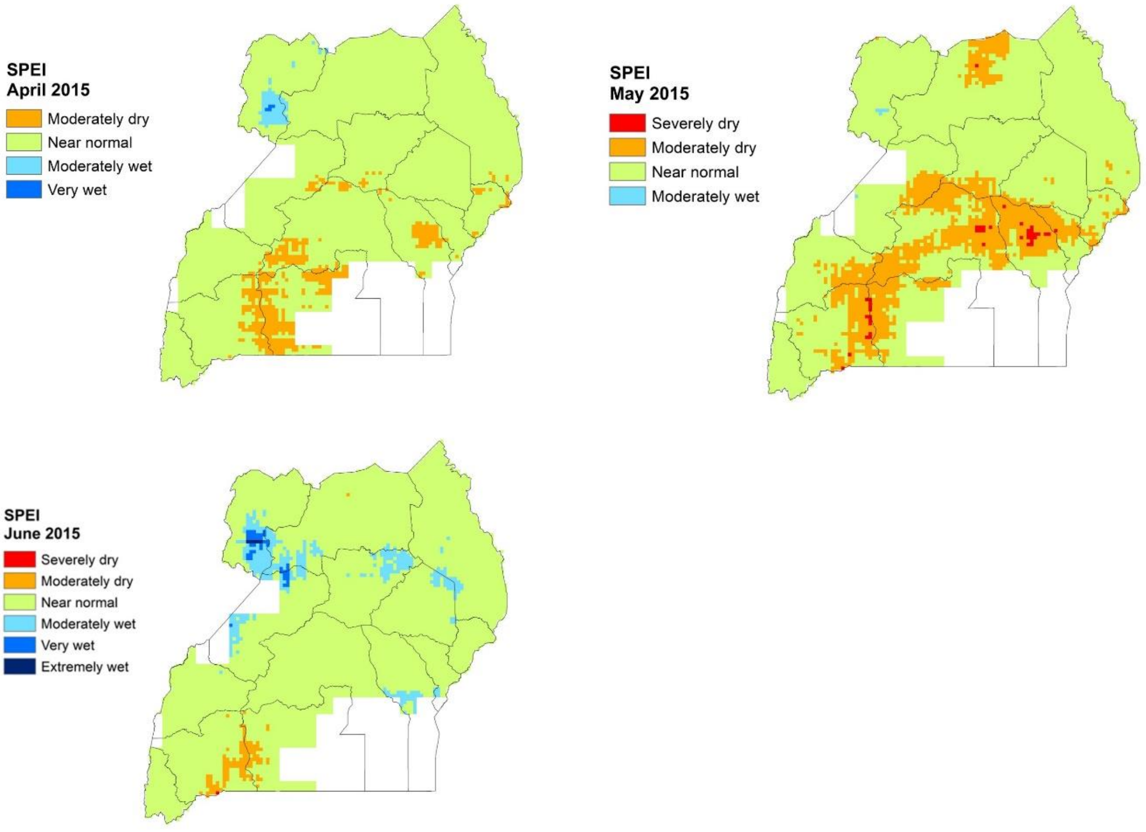

Figure S6. Standardized Precipitation Evapotranspiration Index (SPEI), 6-month accumulation, monthly values for Uganda, for April to June 2015. The SPEI data values have been computed for Africa at 5km resolution, monthly, for the period 1981 to 2016 (Peng et al., 2020).

References

CIESIN (Center for International Earth Science Information Network). Columbia University. Global Gridded Relative Deprivation Index (GRDI), Version 1. Palisades, New York: NASA Socioeconomic Data and Applications Center (SEDAC). (2022a) https://doi.org/10.7927/3xxe-ap97

Emberson, L.D., Ashmore, M.R., Cambridge, H.M., Simpson, D. & Tuovinen, J.P. Modelling stomatal ozone flux across Europe. *Environ. Pollut.*, **109**, 403–413 (2000).

Emmanouil, G., Vlachogiannis, D. & Sfetsos, A. Exploring the ability of the WRF-ARW atmospheric model to simulate different meteorological conditions in Greece. *Atmos. Res.*, **247**, 105226 (2021).

Fick, S.E. & Hijmans, R.J, 2017. WorldClim 2: new 1km spatial resolution climate surfaces for global land areas. *Int. J. Climatol.*, **37**, 4302-4315 (2017).

Fuhrer, J. The critical level for ozone to protect agricultural crops –an assessment of data from European open-top chamber experiments, in: J. Fuhrer, B. Achermann (Eds.), *Critical Levels for Ozone*. UNECE Workshop Report, Schriftenreihe der FAC Berne-Liebefeld, pp. 42–57. (1994)

Harris, I., Osborn, T.J., Jones, P. & Lister, D. Version 4 of the CRU TS monthly high-resolution gridded multivariate climate dataset. *Sci. Data*, **7**,109 (2020).

Hayes, F., Sharps, K., Harmens, H., Roberts, I. & Mills, G. Tropospheric ozone pollution reduces the yield of African crops. *J. Agron. Crop. Sci.*, **206**, 214–228 (2019).

International Food Policy Research Institute (IFPRI), "Spatially-Disaggregated Crop Production Statistics Data in Africa South of the Saharan for 2017", https://doi.org/10.7910/DVN/FSSKBW, Harvard Dataverse, V1 (2020).

Kadaverugu, R., Matli, C. & Biniwale, R. Suitability of WRF model for simulating meteorological variables in rural, semi-urban and urban environments of Central India. *Meteor. Atmos. Phys.*, **133**, 1379-1393 (2021).

Kirenga, B.J. *et al.* 2015. The state of ambient air quality in two Ugandan cities: a pilot cross-sectional spatial assessment. *Int. J. Environ. Res. Public Health*, **12,** 8075-8091 (2015).

Mazzeo, A. *et al.* Evaluation of the WRF and CHIMERE models for the simulation of PM2. 5 in large East African urban conurbations. *Atmos. Chem. Phys.*, **22**, 10677-10701 (2022).

Mills, G. *et al.* Ozone pollution will compromise efforts to increase global wheat production. *Glob. Chan. Biol.*, **24**, 3560-3574 (2018a).

Mills, G. *et al.* Closing the global ozone yield gap: quantification and co-benefits for multi-stress tolerance. *Glob. Chan. Biol.*, **24**, 4869–4893 (2018b).

Mitheu, F. *et al.* The utility of impact data in flood forecast verification for anticipatory actions: Case studies from Uganda and Kenya. *J. Flood Risk Manag.*, e12911 (2023).

Okure, D. *et al.* Characterization of ambient air quality in selected urban areas in Uganda using low-cost sensing and measurement technologies. *Environ. Sci. Tech.*, **56**, 3324-3339 (2022).

Pandey, D. *et al.* Assessing the costs of ozone pollution in India for wheat producers, consumers, and government food welfare policies. *Proc. Natl. Acad. Sci. USA*, **120** (32) (2023).

Peng, J. *et al.* A pan-African high-resolution drought index dataset. *Earth Syst. Sci. Data*, **12**, 753-769 (2020).

Simpson, D. *et al*. The EMEP MSC-W chemical transport model - technical description. *Atmos. Chem*. *Phys*., 12: 7825-7865 (2012).

Stohl, A. *et al.* Evaluating the climate and air quality impacts of short-lived pollutants. *Atmos. Chem. Phys.,* **15**, 10529-10566 (2015).

Uganda Bureau of Statistics (UBOS). *Uganda census of agriculture 2008/2009*. Kampala, Uganda. (2010). https://www.ubos.org/wp-content/uploads/publications/03_2018UCACrop.pdf

Uganda Bureau of Statistics (UBOS). *Uganda annual agricultural survey 2018*. Kampala, Uganda. (2020). https://www.ubos.org/wp-content/uploads/ publications/ 06_2020AAS_2018_Report_Final_050620.pdf
